# Beyond the Primary Infarction: Focus on Mechanisms Related to Secondary Neurodegeneration after Stroke

**DOI:** 10.3390/ijms232416024

**Published:** 2022-12-16

**Authors:** Lin Kooi Ong

**Affiliations:** 1 School of Health and Medical Sciences & Centre for Health Research, University of Southern Queensland, Toowoomba, QLD 4350, Australia; lin.ong@usq.edu.au; 2 School of Pharmacy, Monash University Malaysia, Selangor 47500, Malaysia; 3 School of Biomedical Sciences and Pharmacy, The University of Newcastle, Callaghan, NSW 2308, Australia; 4 Heart and Stroke Research Program, Hunter Medical Research Institute, New Lambton Heights, NSW 2305, Australia

Recently, a growing body of evidence has indicated that secondary neurodegeneration after stroke occurs at remote regions of the brain that are connected to the primary infarction site. For instance, following a middle cerebral artery stroke, secondary neurodegeneration was observed in the ipsilateral thalamus, substantia nigra, white matter tract and hippocampus, despite these regions being remote from the site of primary infarction [1,2]. The neuropathological changes caused by secondary neurodegeneration after stroke that have been documented include brain tissue atrophy, neuroinflammation, blood–brain barrier dysregulation and the accumulation of neurotoxic proteins [3,4]. Secondary neurodegeneration develops within days and continues for months after the initial stroke event and has been suggested as a potential modulator of late phase post-stroke functional disturbances [5,6]. Therefore, the objective of this Special Issue is to expand our understanding of the mechanisms related to secondary neurodegeneration after stroke, as well as the development of therapeutic targets that can ameliorate secondary neurodegeneration, leading to functional recovery. This Special Issue is composed of a communication, three articles and two reviews that are briefly outlined below. 

Accumulated data indicate that neuroinflammation, as demonstrated by the activation of resident inflammatory cells such as microglia and astrocytes, as well as infiltrating peripheral inflammatory cells, is a prominent hallmark of secondary neurodegeneration in pre-clinical and human studies [7,8,9]. Turner and colleagues provided a comprehensive review on neuroinflammatory changes after stroke, particularly at sites of secondary neurodegeneration and anti-inflammatories as potential therapies to target secondary neurodegeneration-associated neuroinflammation [10]. Existing clinical data on immunomodulatory drugs such as Minocycline, Fingolimod, Natalizumab and Interleukin-1 receptor antagonist were discussed in this review. This review introduced the concept of “secondary” neuroprotection for secondary neurodegeneration after stroke given the wider therapeutic window to modulate neuroinflammation for beneficial outcomes. 

Microglia, the primary resident inflammatory cells of the CNS, play an important role in responding to stroke-induced tissue injury within the primary infarction site as well as the sites of secondary neurodegeneration [7,11]. Brown reviewed the contribution of microglial phagocytosis on delayed neuronal death after stroke [12]. Several phagocytic receptors on microglia, including the P2Y_12_ receptor, P2Y_6_ receptor and complement receptor 3, were discussed as potential targets to reduce secondary neurodegeneration after stroke. In a subsequent communication paper, Brown and Milde investigated the role of the microglial phagocytic P2Y_6_ receptor in the prevention of neuronal loss after stroke [13]. Using P2Y_6_ receptor knockout mice, they demonstrated that neurons were spared from microglial phagocytosis, suggesting that inhibition of the P2Y_6_ receptor after stroke may be beneficial to prevent delayed neuronal loss by microglial phagocytosis. 

Stress is known to negatively interfere with rehabilitation and is associated with poorer functional outcomes post-stroke [14]. The hypothalamic–pituitary–adrenal axis is one of the key endocrine systems that regulate stress response, and cortisol is the major stress hormone. Walker and colleagues examined the influence of corticosterone (the rodent equivalent of cortisol, a stress hormone) on neuropathological changes in the white matter tract after cortical photothrombotic stroke [15]. They documented structural changes in the white matter tract along with gliosis after stroke, and these processes were moderately influenced by corticosterone at stress-like levels. 

The enhancement of brain recovery after stroke remains a challenging area, with no therapeutic intervention being widely adopted to promote the recovery of brain function and improve functional outcomes in clinical practice. Clarkson and team investigated the therapeutic potential of glycomimetic compounds along with recombinant human recombinant human brain-derived neurotrophic factor in alleviating post-stroke cognitive impairment [16]. They demonstrated that intracerebral administration of glycomimetic compound and recombinant human-brain-derived neurotrophic factor combination improved post-stroke spatial memory. Furthermore, reductions in stroke-induced reactive astrogliosis and loss of thalamocortical connectivity were observed in this treatment combination. In another study, Hosp and colleagues investigated the therapeutic potential of substance P on post-stroke motor recovery [17]. Systemic administration of substance P facilitated motor recovery and prevented delayed dopaminergic cell loss. These findings provide new evidence about the potential therapeutic effects of recombinant human-brain-derived neurotrophic factor and substance P in stroke recovery. 

Taken together, this Special Issue offers critical insights to the mechanisms related to secondary neurodegeneration after stroke and suggests potential strategies that may help to improve recovery after stroke. Further research in this space is needed to better understand the pathophysiology of secondary neurodegeneration after stroke, and more rigorous pre-clinical studies and human trials should be conducted to validate the potential therapies. 
